# Geospatial analysis of a COVID-19 outbreak at the University of Wisconsin–Madison: potential role of a cluster of local bars

**DOI:** 10.1017/S0950268822000498

**Published:** 2022-04-05

**Authors:** Jeffrey E. Harris

**Affiliations:** 1 Massachusetts Institute of Technology, Cambridge, MA 02139 USA; 2 Eisner Health, Los Angeles, CA 90015 USA

**Keywords:** SARS-CoV-2, networks, mobile device tracking, case control study, instrumental variables

## Abstract

We combined smartphone mobility data with census track-based reports of positive case counts to study a coronavirus disease 2019 (COVID-19) outbreak at the University of Wisconsin–Madison campus, where nearly 3000 students had become infected by the end of September 2020. We identified a cluster of twenty bars located at the epicentre of the outbreak, in close proximity to campus residence halls. Smartphones originating from the two hardest-hit residence halls (Sellery-Witte), where about one in five students were infected, were 2.95 times more likely to visit the 20-bar cluster than smartphones originating in two more distant, less affected residence halls (Ogg-Smith). By contrast, smartphones from Sellery-Witte were only 1.55 times more likely than those from Ogg-Smith to visit a group of 68 restaurants in the same area [rate ratio 1.91, 95% confidence interval (CI) 1.29–2.85, *P* < 0.001]. We also determined the per-capita rates of visitation to the 20-bar cluster and to the 68-restaurant comparison group by smartphones originating in each of 21 census tracts in the university area. In a multivariate instrumental variables regression, the visitation rate to the bar cluster was a significant determinant of the per-capita incidence of positive severe acute respiratory syndrome coronavirus 2 (SARS-CoV-2) tests in each census tract (elasticity 0.88, 95% CI 0.08–1.68, *P* = 0.032), while the restaurant visitation rate showed no such relationship. The potential super-spreader effects of clusters or networks of places, rather than individual sites, require further attention.

## Introduction

Public health officials and researchers have made considerable efforts to pinpoint and understand super-spreader events during the ongoing coronavirus disease 2019 (COVID-19) pandemic [1, 2]. Most of these efforts have focused on the identification of outbreaks at discrete sites with a high concentration of susceptible people, such as assisted living facilities, detention centres, sports arenas, reception halls and food processing plants [3–6]. Other studies have attempted to assess the spillover effects of identifiable mass gatherings, including political rallies [7, 8].

Here, we take a different tack. We focus not on discrete places, but instead on super-spreading clusters or networks of places. We think of infected individuals as moving readily between multiple places within the cluster or network. The component places are linked together by close geographic proximity, or by efficient transportation links.

This network super-spreader model underlies our analysis of the potential role of a local cluster of off-campus bars in a COVID-19 outbreak at the University of Wisconsin–Madison, where nearly three thousand students tested positive for severe acute respiratory syndrome coronavirus 2 (SARS-CoV-2) during September 2020. To address this question, we analyse the geospatial relationships between the movements of smartphones and the distribution of COVID-19 cases. Researchers have increasingly resorted to geospatial techniques to address hard-to-tackle epidemiologic questions [9–15].

Bars have been cited as a potential locus of viral propagation. A retrospective study of infected persons yielded evidence of a contributing role for bar attendance [16]. One smartphone-based study tracked visits to local bars in order to shed light on differences in COVID-19 incidence between Dane and Milwaukee counties during the second wave of the epidemic in Wisconsin [11]. As evidence supporting our model of a network of places, South Korean authorities in May 2020 reported an outbreak of 34 cases after a 29-year-old patient visited five clubs and bars in Itaewon over the course of one night [17, 18].

College and university outbreaks have likewise received attention [19, 20]. One study of a university campus in North Carolina identified multiple clusters of infection in residence halls, athletic teams and fraternities and sororities [21]. Genomic sequencing of SARS-CoV-2 cases in a Wisconsin college outbreak traced the paths of transmission from infected students to vulnerable individuals in the general population [22]. In a preliminary look at 50 counties that contain four-year colleges, coronavirus cases tended to surge 4–12 days after students moved in [23]. In another study of counties containing large colleges or universities, COVID-19 incidence over the 3 weeks before and after the start of classes rose by 56.2% where schools had in-person instruction, but declined by 17.9% where schools had remote instruction [24].

## Methods

### Data: COVID-19 cases

**Fig. 1.**
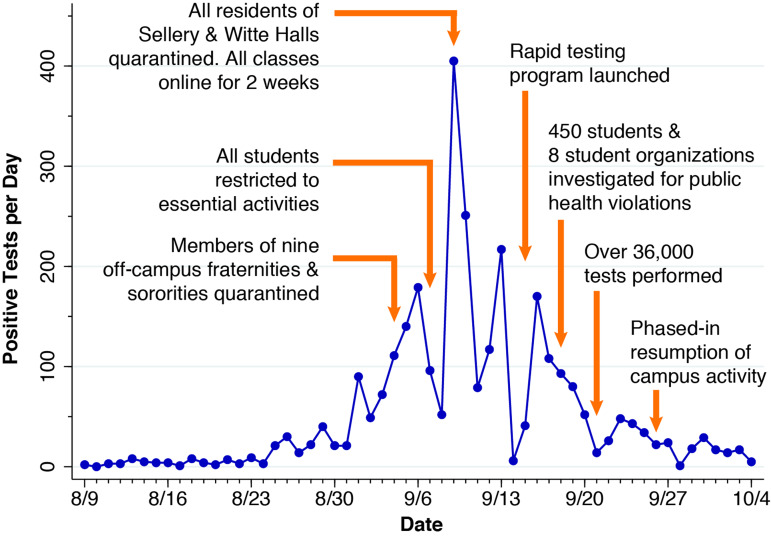
Reported positive SARS-CoV-2 tests per day, University of Wisconsin–Madison students, 9 August – 4 October 2020. The run-up starting in the last week of August culminated in a prominent spike of 656 cases during 9–10 September, followed by a gradual run-off during the remainder of September. In total, 2955 students were documented to be positive during the interval covered by the figure. For further details, see the narrative in [29].

We relied upon the Wisconsin Department of Health Services (WDHS) [25] for data on daily counts of positive SARS-CoV-2 tests by census tract. We cross-checked this source against the daily counts of positive tests reported on the University of Wisconsin–Madison dashboard [26]. Figure 1 relies on the latter source to plot the path of the outbreak during 9 August – 4 October 2020. Superimposed are the principal measures taken by the university. These included a two-week suspension of in-person classes, extensive testing of all students and employees [27], and the quarantine of all residents of two on-campus residence halls, Sellery and Witte, where an estimated 20% of residents had tested positive by the first week of September [28].

### Data: geography, housing and demographics

Screenshots of campus maps, derived from the university's website [30] were overlaid with census tract boundaries derived from the U.S. Census Bureau's TIGER shape files [31] for Dane County, Wisconsin, where the university is located. Additional information on the boundaries of census block groups, which are fully contained within their parent census tracts, was derived from GeoData@Wisconsin [32]. We used QGIS software [33] to locate the centroids of each census tract polygon, and then applied the Haversine formula [34] to compute the distance between centroids, to be used as an instrumental variable in the regression analysis described below.

On-campus residence halls were similarly identified from the university campus map. Data on the number of student residents in each hall, to be used in the case–control study described below, were publicly posted by the university [35]. The estimated 2018 populations of each census tract, used as the denominator to compute case incidence rates, were derived from *datacommons.org* [36] The median income of each census tract, also used as an instrumental variable in our regression analysis, was derived from derived from *statisticalatlas.com* [37].

### Data: bars and restaurants

To identify off-campus bars, we relied upon Google, Yelp and individual websites to compile a list of 51 businesses in the University of Wisconsin–Madison area, variously described as a bar, bar-grill, lounge, pub, tavern or, in some cases, a restaurant, whose principal source of business appeared to be serving alcoholic beverages. We excluded clubs and venues that may have served alcoholic beverages but opened only intermittently for scheduled events. We further excluded those entities that did not match any *point of interest* in the SafeGraph Patterns database, described below. These 51 off-campus bars are mapped in Fig. A in the Appendix. To define the cluster of bars that is the focus of our analysis, we narrowed the list to the 20 bars located in census tracts 16.03, 16.04 and 16.06, as further noted in Fig. A.

To construct a comparison group of restaurants in the same area as the cluster of bars, we again searched all points of interest in the SafeGraph Patterns database that were located in the same census tracts 16.03, 16.04 and 16.06, as well as the extreme eastern end of tract 11.02. Similarly relying on Google, Yelp and individual websites, we isolated a total of 68 entities, including 10 cafés, 37 restaurants rated as inexpensive (including burgers, pizza and ramen), and 21 restaurants rated as moderately priced. We excluded expensive restaurants, wine/liquor stores, smoke and hookah shops, food markets, convenience stores and ice cream parlours.

### Data: smartphone mobility

We relied upon two data sources provided by SafeGraph: the Patterns database [38], and the Social Distancing database [39]. SafeGraph follows the movements of an anonymised panel of smartphones equipped with location-tracking software, where repeated pings from the devices are mapped into their geocoded locations.

The Patterns database, in particular, provides information on the movements of smartphones equipped with location-tracking software to numerous *points of interest* throughout the United States. We relied upon this data source in a study of the role of intrahousehold transmission in the COVID-19 epidemic in Los Angeles County [13].

A separate instalment of the Patterns database [38] is issued each calendar month. Within each monthly instalment, each point of interest has its own record. Within each record, we used the variable *location_name* to identify specific bars and restaurants. We used the variable *visitor_home_cbgs* to identify the home census block groups of all visitors during September 2020, where a device's *home* is the location where it is regularly located overnight. This variable permitted us to compute the number of visitors during September from each census block group to the cluster of 20 bars and to the comparison group of 68 restaurants, where the same device visiting two different bars during September would be counted as two visitors. We further used the variable *visits_by_day* to compute the daily total number of visits to each point of interest for each calendar day in both August and September.

The Social Distancing database [39], by contrast, provides information on both the origin and destination census block groups of device holders. We relied upon this data source in a study of the movements of individuals from their *home* locations to subway stations located throughout New York City during the initial COVID-19 outbreak in February–March 2020 [15].

A separate instalment of the Social Distancing database is issued each calendar day. Within a particular daily instalment, each census block group has its own record. The variable *candidate_device_count* describes the number of devices known to have a home in the census block group, while the variable *completely_home_device_count* gives the numbers of such devices originating in each census block group that stayed completely at home during a particular day. These data allowed us to compute a daily series of the percentage of devices staying completely at home in each census block group. Since SafeGraph updates a device's home census block group at six-week intervals, the devices of many students who had only recently arrived on campus were not captured in the candidate device count. Still, there were sufficient numbers of device homes in the key census block groups in and around the university campus to detect the effects of a university-imposed lockdown.

### Retrospective case–control study

We compared a pair of on-campus residence halls, Sellery and Witte, which we designate as *cases*, to another pair of on-campus residence halls, Ogg and Smith, designated *controls*. As noted above, the Sellery-Witte pair, occupying census block group 16.06-4, was placed on quarantine starting 9 September as cumulative infections reached 1 in 5 residents. By contrast, in the Ogg-Smith pair, occupying census block group 16.06-3, no such lockdown was imposed.

For both the cases and controls, we used the SafeGraph Patterns data to compute the numbers of smartphone visitors during September 2020 to the previously identified cluster of 20 off-campus bars. We denote these counts by *b*_1_ and *b*_0_, respectively. We then computed the ratio *R*_*b*_ = (*b*_1_/*N*_1_)/(*b*_0_/*N*_0_), where *N*_1_ and *N*_0_, respectively, are the known numbers of occupants of the case and control residence halls. The ratio *R*_*b*_ thus represents the relative bar visitation rate of an occupant of the Sellery-Witte case residence halls compared to an occupant of the Ogg-Smith control residence halls. Similarly, we determined *r*_1_ and *r*_0_, the respective numbers of visitors during September to the previously identified comparison group of 68 restaurants. We then computed the analogous ratio *R*_*r*_ = (*r*_1_/*N*_1_)/(*r*_0_/*N*_0_), which represents the relative restaurant visitation rate of occupants of the case and control residence halls. Combining our calculations, we tested the null hypothesis that *R*_*b*_ = *R*_*r*_ or, equivalently, *R*_*b*_/*R*_*r*_ = (*b*_1_/*b*_0_)/(*r*_1_/*r*_0_) = 1. Finally, to assess the influence of spatial proximity, we repeated our case–control analysis on the subset of 4 bars and 12 restaurants that were no more than a 6-minute walk from Sellery-Witte.

### Regression analysis

We formulated regression models relating the per-capita incidence of newly positive coronavirus tests to the per-capita rates of visitation to bars and restaurants across 24 census tracts in the University of Wisconsin–Madison area. Let *y*_*i*_ denote the incidence of newly positive tests per 1000 population reported by WDHS for census tract *i* during September 2020. Let *x*_*bi*_ and *x*_*ri*_, respectively, denote the numbers of devices with a home in census tract *i* visiting the designated bars and restaurants during August–September, divided by the corresponding population of census tract *i*. We used both ordinary least squares (OLS) and instrumental variable (IV) regression to estimate the constant-elasticity model log *y*_*i*_ = *α* + *β*log *x*_*bi*_ + *γ*log *x*_*ri*_ + *ɛ*_*i*_, where *ɛ*_*i*_ are assumed to be i.i.d. normally distributed errors. For IV estimation, our two instruments were: the distance between the centroid of census tract *i* and the centroid of census tract 16.06, where the cluster of bars and restaurants was located; and the median income of each census tract *i*. In both OLS and IV specifications, we tested the null hypotheses that *β* = 0 and *γ* = 0. To test for spatial non-stationarity in our data, we also ran our constant-elasticity model under geographically weighted regression (GWR) [40, 41].

## Results

### Descriptive mapping

**Fig. 2.**
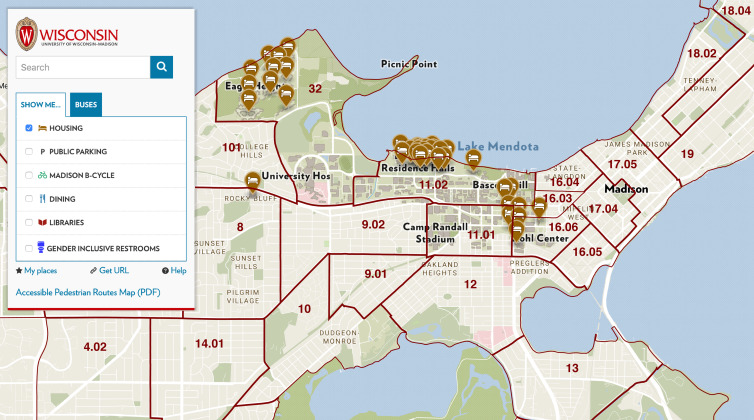
University of Wisconsin–Madison Campus Map with Locations of On-Campus Housing, with Overlaid Census Tract Boundaries. The campus occupies all of census tracts 32 and 11.02, and parts of tracts 11.01, 16.03 and 16.06. The residential halls are located principally in census tracts 32, 11.01, 11.02 and the west end of 16.06. To the east, we see the Capitol neighbourhood of Madison, including tracts 16.03, 16.04, 16.05, 17.04 and 17.05.

Figure 2 shows the distribution of on-campus residential facilities within the university campus. The solid, wine-coloured lines mark the superimposed boundaries between census tracts. Appendix Fig. B shows the locations of off-campus fraternities and sororities, while Appendix Fig. C shows the locations of off-campus housing in the vicinity of the university.

**Fig. 3.**
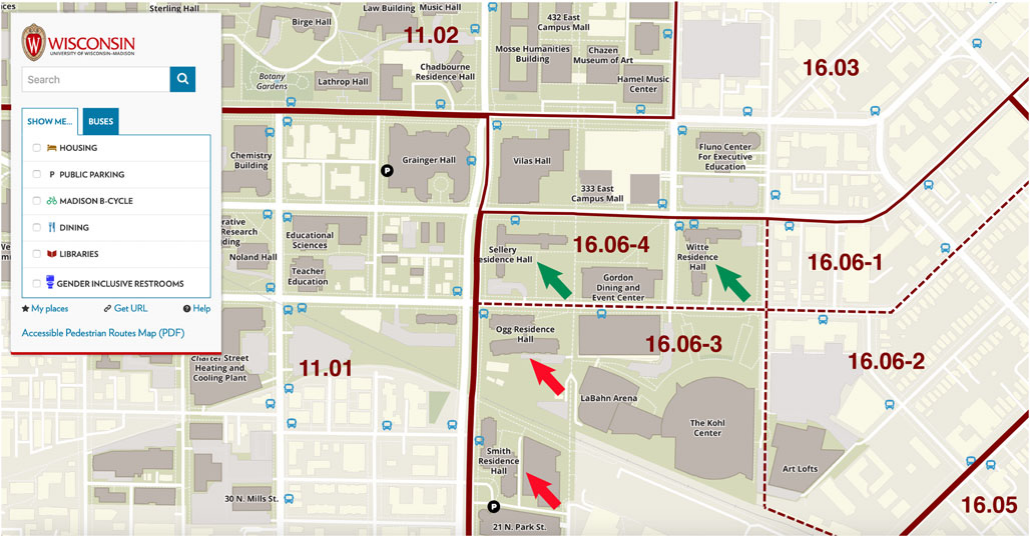
Details of U. Wisconsin–Madison campus map, highlighting Sellery and Witte Residence Halls (green arrows) within census block group 16.06-4 and Ogg and Smith Residence Halls (red arrows) within census block 16.06-3. Sellery and White were the only residential structures located within census block group 16.06-4. With the exception of two small buildings with a commercial tenant on the first floor and limited space for a handful of residential apartments on the second floor [42], Ogg and Smith were the only residential properties in 16.06-3.

In Figure 3, we have zoomed down on the university campus map, focusing on the eastern end of the campus. As in Figure 2, the superimposed solid, wine-coloured lines mark the external boundaries between the census tracts. Here, the dashed lines also separate the internal boundaries between the four census block groups within tract 16.06. The green arrows locate Sellery and Witte Residence Halls, the two residence halls subject to quarantine during the peak of the outbreak. To the south, in census block group 16.06-3, the red arrows identify Ogg and Smith Residence Halls, where no lockdown was imposed. Sellery and White were the only residential structures located within census block group 16.06-4, and with minor exception, Ogg and Smith were the only residential properties in 16.06-3. As result, we could reliably conclude that any smartphone visits homed in census block group 16.06-4 were residents of Sellery and White, while any smartphone visits homed in census block group 16.06-3 were residents of Ogg and Smith.

### Social distancing data

**Fig. 4.**
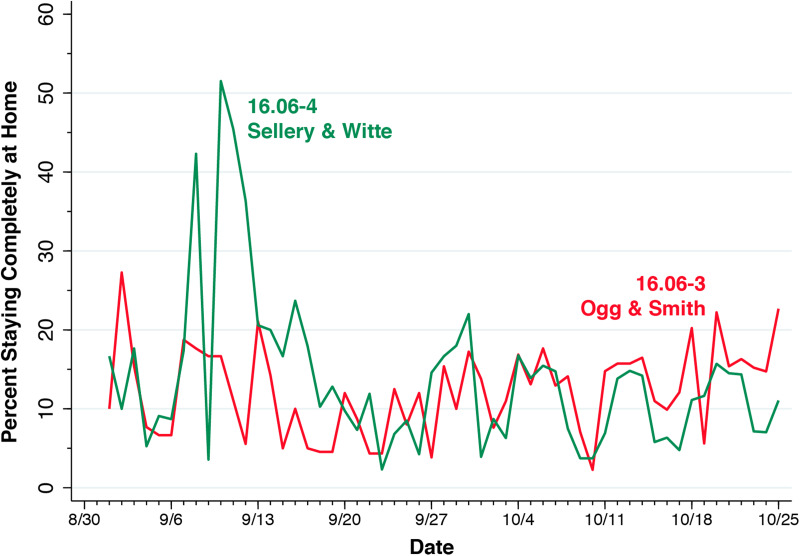
Percentage of devices staying completely at home in census block groups 16.06-3 and 16.06-4. The spike in the percentage of devices staying completely at home did not last the entire 2-week lockdown. Students in Sellery-Witte were subsequently allowed to leave their dorms for 15–20 min to eat at the nearby Gordon Dining and Event Center, identified in Figure 3 above.

Figure 4 shows the proportion of smartphone devices staying completely at home among all devices whose *home* was identified as census block group 16.06-3 or 16.06-4, based upon the SafeGraph Social Distancing data. For census block group 16.06-4, which captures residents of Sellery and Witte, the figure shows a spike in the proportion of devices staying completely at home on 8 September, a finding compatible with voluntary self-quarantining as the proportion infected in these dorms neared 20%. There followed an abrupt drop on 9 September, a finding consistent with a last-minute escape before the pending lockdown went into effect at Sellery and Witte that evening at 11 p.m. The university-imposed quarantine at these two residence halls is captured by the second spike on 10 September, followed by a sustained increase in stay-at-home devices during the next week. No such patterns were seen among residents of Ogg and Smith in census block group 16.06-3.

### COVID-19 cases

**Fig. 5.**
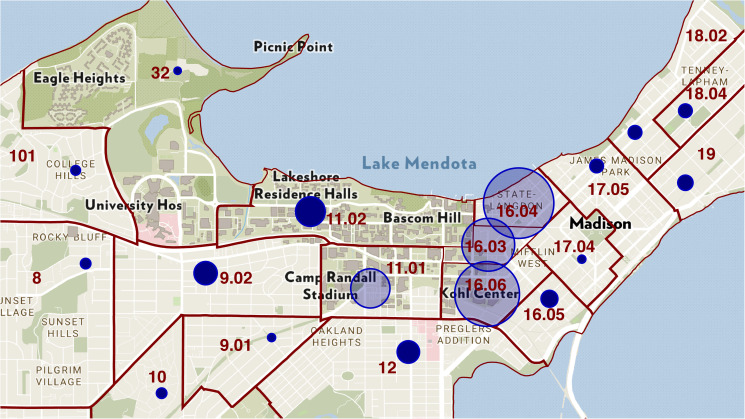
Map of cumulative COVID-19 positive cases during 16 August – 16 October in relation to census tract in the U. Wisconsin–Madison area, as reported by the WDHS [25]. The cumulative number of cases is proportional to the area of each bubble.

Figure 5 maps the cumulative number of COVID-19 positive cases diagnosed during the 2-month period from 16 August through 16 October 2020 in each of the census tracts surrounding the U. Wisconsin–Madison campus, as compiled by the WDHS [25]. The cumulative number of cases is proportional to the area (not the diameter) of each bubble. The four largest bubbles are: tract 16.04 (870 cases); tract 16.06 (726 cases); tract 16.03 (488 cases); and tract 11.01 (266 cases). These four census tracts comprised 76.7% of the 3065 cases in campus-area census tracts compiled by the WDHS.

**Fig. 6.**
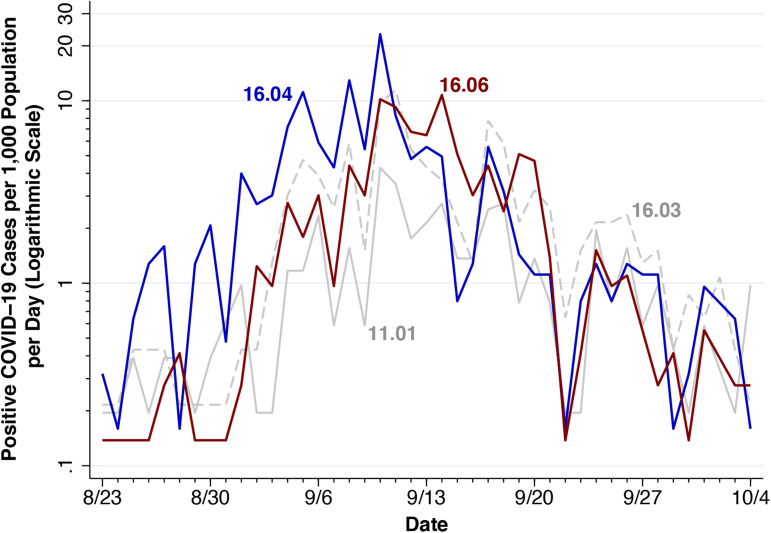
Positive COVID-19 cases per 1000 population per day in four key census tracts in Madison WI, 23 August – 4 October 2020. We have coloured the paths for tracts 16.03 and 11.01 in light grey to help elucidate the epidemic paths in the two principal tracts, 16.04 and 16.06. The incidence is measured on a logarithmic scale to show relative changes.

Figure 6 elucidates the dynamics of COVID-19 incidence in these four census tracts. Plotted are the daily incidence rates per 1000 population from 23 August – 4 October. The figure suggests that there were, in fact, two distinct waves to the outbreak. The first wave originated in tract 16.04, where we have identified a high concentration of fraternities and sororities (Appendix Fig. B) and off-campus housing generally (Appendix Fig. C). By the last week of August, daily incidence had already crossed to 1-per-1000 threshold, about four times the daily rate in Dane County during the peak of the second epidemic wave in early July [11]. By 2 September, as noted above, an outbreak had first been detected in nine off-campus fraternities. The incidence during this first wave peaked at 23 per 1000 on 10 September.

The second wave, concentrated in tract 16.06, lagged behind the first wave by about 4–5 days, but grew more rapidly. The rise in incidence from 0.28 to 10.19 per 1000 during September 1–10 implies a reproductive number of about 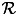 = 2.6 [43]. While tract 16.06 included on-campus residence halls and off-campus housing (Fig. 2 and Appendix Fig. C), Figure 6 is compatible with the 9 September lockdown of Sellery and Witte Residence Halls.

### Smartphone device movements

**Fig. 7.**
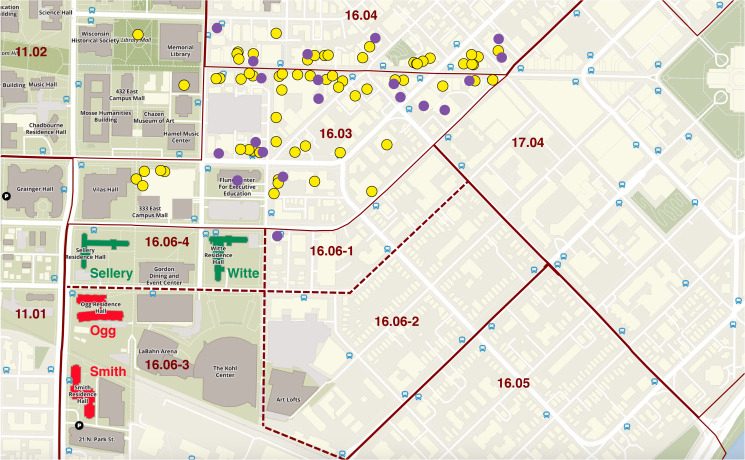
Section of U. Wisconsin–Madison campus map, with census tract and census block group boundaries, locations of four residence halls, a cluster of 20 nearby off-campus bars (purple), and 68 comparison restaurants (yellow).

Figure 7 superimposes the locations of the cluster of 20 off-campus bars (solid purple circles) and the comparison group of 68 coffee houses, inexpensive and medium-priced restaurants (yellow-filled circles) on a section of the university campus map. As in Figure 3, we have identified Sellery and White (green buildings) in census block group 16.06-4 and Ogg and Smith (red buildings) in census block group 16.06-3.

**Fig. 8.**
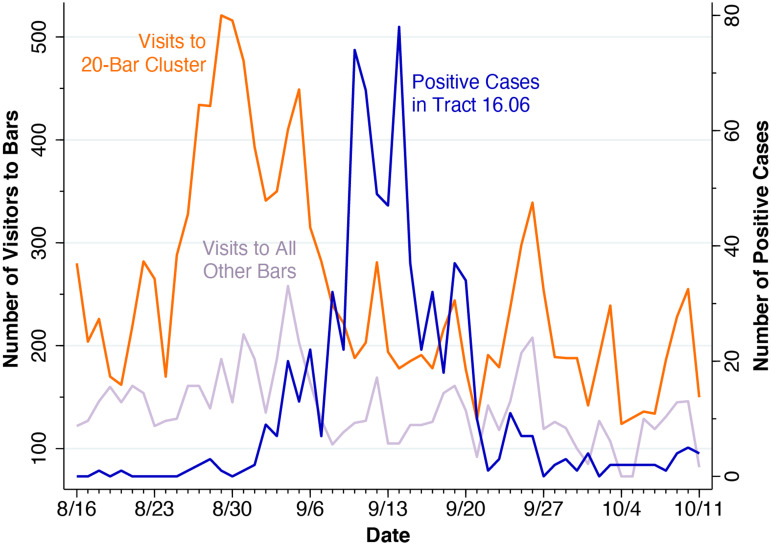
Orange series: Daily Visits by Device Holders to Bars Within the 20-Bar Cluster Identified in Figure 7. Lilac: Daily Visits by Device Holders to the 31 Other Off-Campus Bars Identified in Appendix Fig. A. Blue: Daily Positive COVID-19 Cases in Census Tract 16.06, as Reported by WDHS.

Figure 8 below contains three concurrent plots covering each day from 16 August through 11 October. The orange line, measured on the left vertical axis, shows the total number of daily visits by all devices, without restriction on origin, to any one of the bars within the 20-bar cluster identified in Figure 7. The lilac line, also measured on the left axis, similarly shows total daily visits to all 31 remaining off-campus bars outside the cluster, which are identified in Appendix Fig. A. These two data series, derived from the Patterns variable *visits_by_day*, capture all visits to bars, regardless of origin, including those recently arrived device holders whose home location has not yet been updated.

Since the Patterns database tracks the GPS pings emitted from a limited panel of devices, only the relative changes in visit counts have significance. Thus, the orange series of visits to nearby bars shows an average of 220 daily visits among device-holders during the week of 16 August, followed by a peak of daily visits to 521 on 29 August, a relative increase of 2.4-fold. By contrast, the lilac series of visits to the remaining off-campus bars showed only a 1.3-fold increase during the same time period. Moreover, the orange series shows a double-peaked surge in the volume of nearby bar attendance, with the first peak occurring on 29–30 August and the second on 5 September. This observation is further consistent with the evidence on voluntary self-rationing by residents of Sellery and Witte seen in Figure 4.

The orange double-peaked surge in nearby bar attendance was followed by a blue double-peaked surge in positive SARS-CoV-2 cases in census tract 16.06, as measured on the right vertical axis in Figure 8. The first peak occurred on 10 September, while the second occurred on 14 September. With WDHS-reported cases lagging one day behind the university's dashboard-reported cases, the data in Figure 8 would be consistent with an incubation period between infection and the development of symptoms in the range of 3–6 days [44].

### Case–control study

Table 1 below shows the data on the numbers of visitors from the two census block groups to all 20 bars and all 68 comparison restaurants in our analysis. The first row corresponds to the *case* residence halls in census block group 16.06-4, while the second row corresponds the *control* residence halls in census block group 16.06-3. The column labelled *N* shows the census of each residence hall pair, where *N*_1_ = 2392 and *N*_0_ = 1198. With *N*_1_/*N*_0_ = 2.01, we see that Sellery-Witte had twice as many residents and Ogg-Smith.

**Table 1.** Case–control calculations^a^

Census block group	*N*	*b*	*r*
16.06-4 (1)	2392	255	426
16.06-3 (0)	1198	43	137
*R*		2.95	1.55

a Estimated odds ratio *R*_*b*_/*R*_*r*_ = 1.91 (95% CI 1.29–2.85).

The column labelled *b* shows the number of visitors to the cluster of 20 bars originating from the two census block groups in September. With *b*_1_/*b*_0_ = 5.93, we see that Sellery-Witte had nearly six times as many bar visitors as Ogg-Smith. Corrected for the difference in census, the ratio is *R*_*b*_ = (*b*_1_/*N*_1_)/(*b*_0_/*N*_0_) = 2.95. The column labelled *r* shows the number of visitors to the comparison group of 68 restaurants in September. With *r*_1_/*r*_0_ = 3.11, we see that Sellery-Witte had about three times as many restaurant visitors as Ogg-Smith. Corrected for the difference in census, the ratio is *R*_*r*_ = (*r*_1_/*N*_1_)/(*r*_0_/*N*_0_) = 1.55. Based on these data, we can then compute the overall ratio *R*_*b*_/*R*_*r*_ = 1.91. This quantity is equivalent to an odds ratio in a classic epidemiologic study, with the bar visitors interpreted as the exposed subjects and the restaurant visitors interpreted as the unexposed subjects. The conditional likelihood estimate of the 95% confidence interval (CI) for this ratio [45] was 1.29–2.85. The null hypothesis that *R*_*b*_/*R*_*r*_ = 1 was rejected at the significance level *P* = 0.0007.

Appendix Table A shows the corresponding case–control calculations for the subset of bars and restaurants within a 6-minute walk of Sellery and Witte. The estimated odds ratio increased to *R*_*b*_/*R*_*r*_ = 2.60, with a 95% CI 1.47–4.66, while the null hypothesis of *R*_*b*_/*R*_*r*_ = 1 was rejected at the significance level *P* = 0.0004. Comparing Table 1 with Appendix Table A, we see that bars located within the 6-minute radius were the destination in 68.1% of visits from the residence halls to the 20-bar cluster. By contrast, restaurants located within the 6-minute radius were the destination in only 28.2% of visits from the residence halls to the 68-restaurant comparison group.

### Regression analysis

**Fig. 9.**
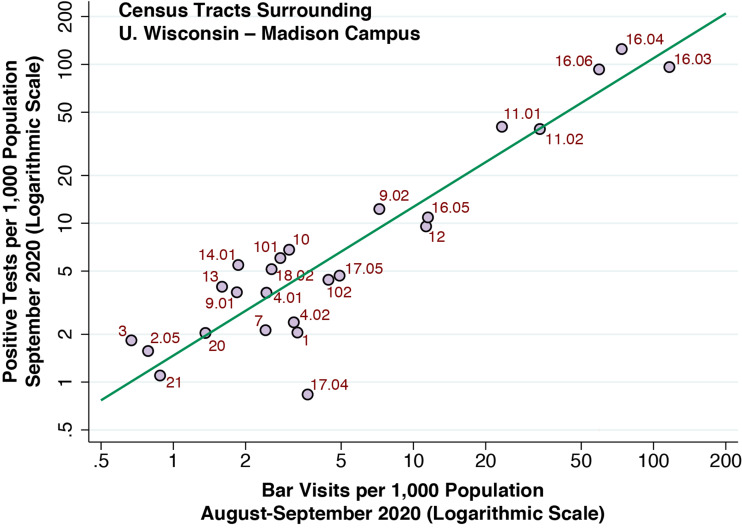
Incidence of positive SAR-CoV-2 tests per 1000 population versus visits per 1000 population to the 20-bar cluster, August–September 2020. The plot displays 21 census tracts in the university area. The fitted line is based on IV regression, where the instrument was the distance from each census tract to bar cluster in tract 16.06.

Figure 9 shows a log-log bivariate plot of the incidence of newly diagnosed positive coronavirus tests per 1000 population *vs.* visits to the cluster of 20 bars per 1000 population during September 2020. The incidence data are broken down by the census tract, while the numbers of bar visits are broken down by the census tract of origin of the bar visitor. The slope of the plotted log-log relationship, estimated via IV regression, was 0.90 (95% CI 0.68–1.13, *R*^2^ = 0.85). The corresponding OLS estimate was 0.91 (95% CI 0.72–1.09, *R*^2^ = 0.85).

Appendix Fig. D shows the corresponding bivariate relationship between coronavirus incidence and per-capita visits to the comparison group of 68 restaurants. Although there was a positive relationship (IV-estimated elasticity 0.88, 95% CI 0.57–1.19), the bivariate plot showed substantially more dispersion and reduced explanatory power (*R*^2^ = 0.64).

Table 2 shows the estimated multivariate relation between coronavirus incidence and per-capita visits to the cluster of 20 bars and the comparison group of 68 restaurants. The estimated coefficients (*β*) for bar visits were statistically significant in both the OLS method (1.21, 95% CI 0.72–1.71, *P* < 0.001) and IV method (0.88, 95% CI 0.08–1.68, *P* = 0.032), while the estimated coefficients (*γ*) for restaurant visits were not. Separate scatterplots showed that both instruments (distance travelled and median income) had strong negative relationships with bar and restaurant visitation rates.

**Table 2.** Regression estimates^a^

Estimation method^b^	*α*	*β*	*γ*
OLS	0.717 (0.274)	1.215 (0.236)	−0.303 (0.217)
IV	0.433 (0.336)	0.880 (0.409)	0.024 (0.352)

a Estimates of the model log *y* = *α* + *β*log *x*_*b*_ + *γ*log *x*_*r*_, where *y* is the incidence of positive coronavirus tests per 1000 population in September 2020, and *x*_*b*_ and *x*_*r*_ are, respectively, the numbers of bar and restaurant visits per 1000 population during August–September 2020. Number of observations = 21 census tracts.

b OLS, ordinary least squares; IV, instrumental variables estimation. Standard errors are in parentheses beside each parameter estimate.

Finally, geographically weighted regression (GWR) revealed no significant evidence of spatial non-stationarity (significance levels *P* = 0.87 for the intercept term, *P* = 0.91 for the log *x*_*b*_ term, and *P* = 0.98 for the log *x*_*r*_ term). The spatial correlation coefficient of the residuals from the OLS model was −0.0054.

## Discussion

### Novel epidemiology

In a conventional case–control study, we would have interviewed infected students (the *cases*) and uninfected students (the *controls*) in order to retrospectively reconstruct their recent past visits to bars and restaurants (that is, the *exposures* of the cases and controls). In this study, by contrast, our subjects were smartphone devices. The cases were those devices homed in a pair of residence halls (Sellery-Witte) known to have been hard hit by COVID-19 infections, while our controls were devices homed in another pair of residence halls (Ogg-Smith) that were not so affected. We ascertained their respective exposures by anonymously tracking the pings of these devices at bars and restaurants. This is, in a genuine sense, a novel twist on one of the classic methodological tools of epidemiology [46].

Pursuing this novel approach, we identified a cluster of 20 bars occupying those census tracts with the highest per-capita incidence rates during the September 2020 outbreak (Figs 5, 7 and Appendix Fig. A). This cluster was in close proximity to the two on-campus residence halls where cumulative infections approached 1 out of 5 occupants and a mandatory quarantine was imposed (Figs 3, 7). Examining device stay-at-home patterns during the quarantine period (Fig. 4), we confirmed that the cases and controls could be identified by those devices homed, respectively, in two census block groups (16.06-4 and 16.06-3).

While the overall daily incidence of newly diagnosed infections peaked on 9–10 September (Fig. 1), we found that the outbreak in fact had two successive waves (Fig. 6). The first wave was dominated by cases from census tract 16.04, where nearly all off-campus fraternities and sororities were located (Fig. B). The second wave, which peaked about 4–5 days later, was dominated by cases from census tract 16.06, where the two on-campus residence halls with the highest infection rate were located (Figs 3, 7). What's more, visits to those bars within the 20-bar cluster directly preceded the upsurge in the incidence of infection with a lag of 3–6 days (Fig. 8), an interval consistent with the known incubation period of the virus [44].

Our case–control study revealed that a resident of Sellery-Witte had a 2.95-fold greater rate of visitation to the entire 20-bar cluster than a resident of Ogg-Smith, which was more distant from the cluster (Table 1). By contrast, a resident of Sellery-Witte had only a 1.55-fold greater rate of visitation to a comparison group of 68 restaurants located in the same off-campus area (Table 1). This gradient between bar and restaurant visitation rates was even more pronounced when we restricted our case–control comparison to points of interest within a 6-minute walk of Sellery-Witte (Appendix Table A). Accordingly, while geographic proximity was a significant determinant of restaurant visitation, it was an even more important determinant of bar visitation.

### Questions of self-selection

Our study, while novel in its methodology, is nonetheless observational. We did not conduct an experiment in which subjects are randomly assigned to visit bars or restaurants to see who comes down with COVID-19. Accordingly, it is at least arguable that people who go to bars are less likely to wear masks, maintain social distancing, and take protective measures generally. The same criticism could be applied to a study of people who attended a political rally, a motorcycle rally, or a large wedding reception. Still, our comparison of residence halls – rather than individuals – tends to blunt this criticism.

Numerous factors go into a student's decision to live in one residence hall *vs.* another: whether the rooms are singles or doubles, whether there is more than one bathroom on a floor, whether the floors are mixed coed, whether the rooms have carpeting or air conditioning, and whether the student can cohabit with his or her friends, not to mention the price. It would be a stretch to argue that these factors readily correlate with a lack of protective behaviour. Sellery and Witte were regarded by some observers as party dorms, at least during prior semesters [47]. This raises the possibility that some smartphone visits to the 20-bar cluster were to purchase alcoholic beverages to bring back to residence hall parties. But that would not negate the causal role of the bars in facilitating such parties.

### Distance as an instrumental variable

In our regression analysis (Fig. 9, Table 2 and Appendix Fig. D), we studied the relation between bar visitation and COVID-19 risk on a much wider geographic scale. To that end, we employed two instrumental variables: the distance from each census tract to the 20-bar cluster; and the median income of each census tract. Both these instruments displayed strong negative relationships with bar and restaurant visitation rates. The strength of median income as an instrument should come as no surprise, as the inverse relation between income and the demand for inexpensive, fast food is well established [48].

Still, the validity of each instrument hinges on an untested assumption that there is a pathway connecting the instrument and the putative causal variable (bar or restaurant visitation), but no such pathway connecting the instrument to the response variable (COVID-19 incidence) [49, 50]. The consistency of the IV estimates of the elasticity of COVID-19 incidence with respect to bar visitation in a simple bivariate model (0.90, 95% CI 0.68–1.13) and a multivariate model (0.88, 95% CI 0.08–1.68) support the validity of this assumption.

The role of distance as an instrumental variable helps further interpret the results of our case–control study. If there is any exogenous factor that clearly distinguishes Sellery and Witte from Ogg and Smith, it is that one pair of residence halls is nearer and the other pair is farther away. But that begs the question: Nearer to or farther from what? The case–control findings support the conclusion that the residents of Sellery and White suffered significantly more infections than the residents of Ogg and Smith because they were nearer to the 20-bar cluster, and not because they were also nearer to coffee houses, inexpensive and moderately priced restaurants, or nearer to some classrooms.

### Why a cluster of places could amplify a disease outbreak

There are important theoretical reasons why a cluster or network of sites may amplify an infectious disease outbreak in comparison either to a disparate collection of independent sites, or to one large site with the same capacity.

First, there is the basic issue of proximity. If Bar A were located in Lower Manhattan while Bar B were located along the Champs-Elysees, to take an extreme example, we can effectively ignore the possibility that patrons of Bar A will later hop over to Bar B. Proximity within a geographic cluster or along a network permits mixing between sites. That's what happened in the above-cited example from South Korea [17, 18].

Second, clusters or networks may take advantage of important non-linearities. In the language of the classic SIR model [51], these non-linearities constitute violations of the mass-action assumption that the incidence of new infections is proportional to the product of *S*, the number of susceptible individuals, and *I*, the number of infective individuals. Instead, the incidence of new infections at any single site may be an increasing but concave function of the intensity of mixing of susceptible and infective persons at that site. Let's say an infective individual *I* sits close to susceptible person *S_A_* in Bar A long enough to infect *S_A_*. If individual *I* stays the rest of the evening talking to *S_A_*, no one else is infected. But if individual *I* decides to get up and go down the street to Bar B, then *I* will have time to infect another person *S_B_* as well.

Third, clusters or networks may create positive externalities. Proximity alone may allow the same individuals to mix in both Bar A and Bar B. But positive externalities go one step further. Consider a group of university students contemplating going out to a bar. ‘Let's go to Bar A’, proposes one of them. Endorsing the first student's proposal, another student adds, ‘And if we can't get into Bar A, we'll go over to Bar B’. In this example, the mere availability of Bar B as an alternative enhances the demand for Bar A.

Fourth, there is the issue of selective mixing. In a sufficiently rich theoretical model, individuals will vary in their infectivity, and infectivity may in turn be positively correlated with mobility [52]. In the context of a cluster or network of bars during a COVID-19 outbreak, asymptomatic infected individuals tend to bar-hop the most.

### We did not demonstrate a network effect

While we used the terms cluster and network interchangeably, we did not have sufficient data in this study to map out the individual connections between places. More concretely, we focused on a cluster of 20 bars at the epicentre of the outbreak, but we had no data on bar-hopping. While the publicly available data from SafeGraph record device movements from one location to another, the device's home is necessarily the origin or the destination. This constraint limits the interpretation of our smartphone movement data. Let's say that on a particular day in late August, ten devices homed in census block group 16.06-4 visited Bar A and ten devices homed in the same census block group visited nearby Bar B. It is possible that the two sets of devices were entirely nonoverlapping. But it is also possible that the same ten devices first went to Bar A and then hopped over to Bar B.

We treated a visit to any one of the 20 bars as a distinct exposure from a visit to any one of the 68 restaurants. Yet nothing in the SafeGraph device-movement data excludes the possibility of hopping between restaurants and bars. This raises the possibility of a network externality between restaurants and bars. Visitors to Restaurant C do not necessarily get infected while dining there. Yet the mere availability of Restaurant C may enhance the demand for Bar D, where patrons do, in fact, get infected.

What we do know from our case–control findings is that the enhanced visitation rates from census block group 16-06.4 were observed in multiple bars in the cluster, and not just a single bar. We also know from anecdotal reports that bar-hopping has long been a tradition in downtown Madison [53–55]. It would be inappropriate to assume that, in absence of hard probative data, each establishment in the 20-bar cluster had no more than an independent effect on the risk of coronavirus propagation.

### Who was student zero?

We identified two distinct waves of the outbreak: a fraternity-sorority-based outbreak localised in census tract 16.06 and an on-campus residence hall-based outbreak localised to census tract 16.04. The upswing of this second wave had an estimated reproductive number of 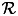 = 2.6. This distinction alone does not inform us whether the first wave seeded the second or, alternatively, that the two waves resulted from multiple distinct importations. Distinguishing between these two hypotheses would ordinarily require phylogenetic analysis of viral samples [15, 22, 56]. Because SafeGraph updated the assigned *home* of many devices on 1 September, many students arriving on campus at the end of August were not immediately reclassified as holders of devices originating in one of the key census block groups. This delay limited our ability to study the early movements of students between the dorms (census block groups 16.06-4 and 16.06-3) and fraternities and sororities (census block groups 16.04-1 and 16.04-4). In any event, it would be premature to conclude that the University of Wisconsin–Madison outbreak originated with a ‘student zero’.

### Perspectives

Our findings should not be interpreted broadly to mean that restaurants are entirely free of risk while bars are the sole source of contagion. The narrower interpretation is that a specific, centrally located cluster of bars appeared to be a significantly greater vehicle for propagation of the virus than restaurants in a particular university-based outbreak. Neither do our findings point the finger at all bars. Among the 51 bars throughout the campus area, we focused sharply on a cluster of 20 bars at the geographic epicentre of the outbreak (Appendix A). Nor should our findings be taken to absolve other high-density conditions that may enhance person-to-person transmission.

Future studies of college and university outbreaks need to concentrate harder on the dynamics of viral transmission, and not simply on how many cases ended up in dormitories, athletic teams, fraternities and sororities [57]. Retrospective case-tracking needs to expand its scope to ask an infected individual not just whether he went to a bar, but also whether he went bar-hopping, whether his roommates also went to bars, and if so, to what bars on what nights. With most large universities and many smaller colleges located in urban environments, the relationship between the university and local authorities may be critical to the prevention and control of outbreaks.

Even more broadly, we need to think about systemic factors that influence viral propagation, and not simply the characteristics of individuals or the places they go to. The epidemic in Los Angeles County has been sustained in great part by intra-household transmission among multigenerational families [13]. But the larger question is what public policies have enhanced or mitigated these housing conditions. The spread of coronavirus in New York City and other metropolises may have been enhanced by individuals of high mobility [52]. But the larger question is what transportation networks carried them from one place to another [15, 58–60].

## Figures and Tables

**Fig. A.**
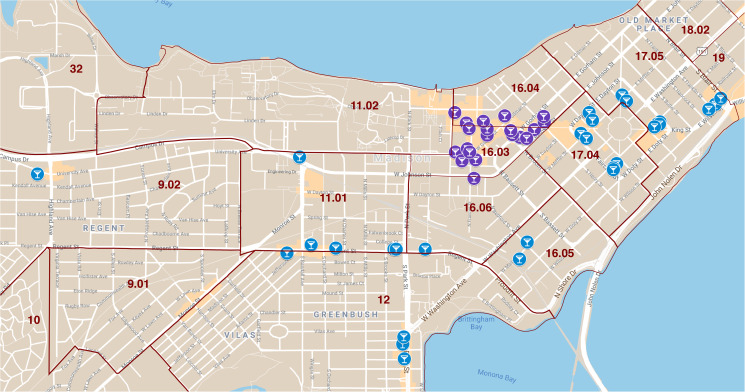
Google My Maps screenshot of 51 bars in the U. Wisconsin–Madison area. The purple markers locate the 20 bars belonging to the campus cluster within census tracts 16.03, 16.04 and 16.06. Among the remaining 31 bars identified by the blue markers, there is a cluster of 10 establishments surrounding the Capitol Square in tracts 17.04 and 17.05, and a separate array of five bars running along Regent Street, bounding tracts 12, 11.01 and 16.06.

**Fig. B.**
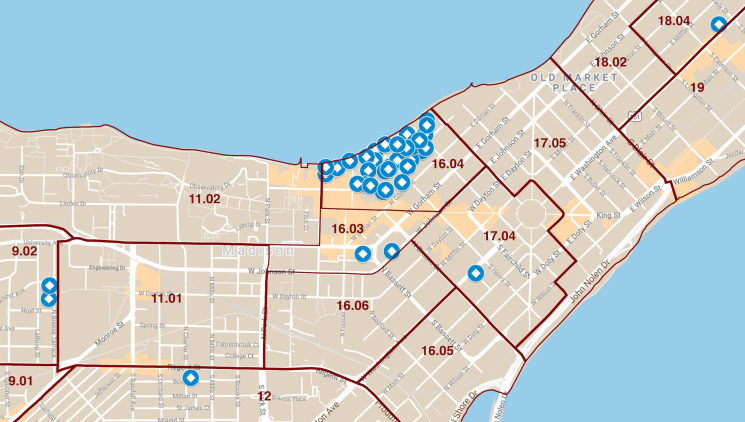
Google My Maps screenshot of the locations of fraternities and sororities in the University of Wisconsin–Madison Area. Among the 43 organisations identified on the university's website [61] with identifiable addresses, 36 had addresses within census tract 16.04.

**Fig. C.**
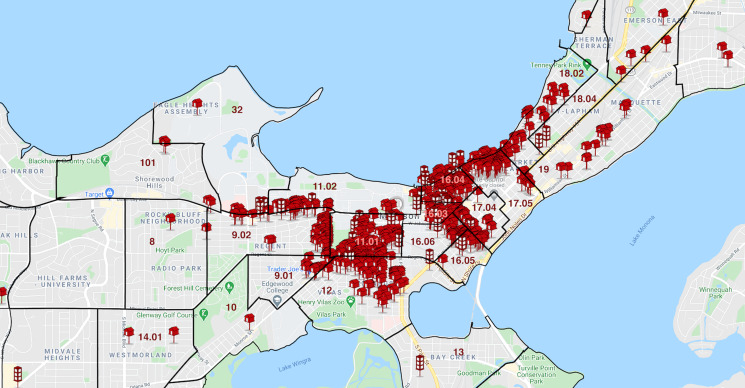
Screenshot of the locations of off-campus housing from U. Wisconsin–Madison off-campus housing marketplace [62], as of 25 October 2020. The map shows the highest concentrations of off-campus housing in tracts 11.01, 12, 16.03, 16.04 and 17.05.

**Fig. D.**
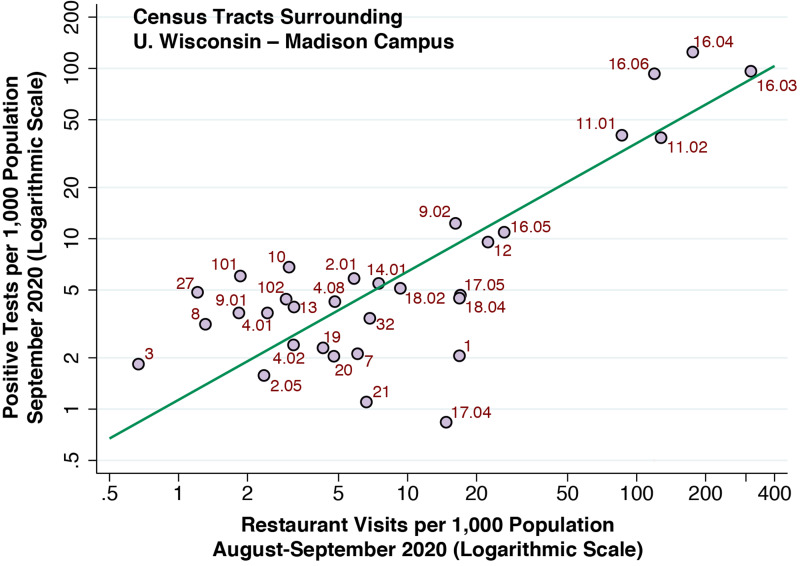
Incidence of positive SAR-CoV-2 tests per 1000 population versus visits per 1000 population to the 68-restaurant group, August–September 2020. The plot displays 24 census tracts in the university area. The fitted line (elasticity 0.88, 95% CI 0.57–1.19, *R*^2^ = 0.65) is based on IV regression, where the instrument was the distance from each census tract to tract 16.06.

## Data Availability

Supporting programs and data have been posted at DOI: 10.17605/osf.io/7cvyh.

## Appendix

### Fraternities and sororities

Among all fraternity and sorority organisations referenced on the university's website [61], we searched Google to find 43 organisations with identifiable addresses in the campus area, whether or not they had communal live-in facilities. We investigated whether an analogous case–control design could be applied to the census block groups where fraternities and sororities were located, particularly within census tract 16.04. Based upon the proportion of devices staying completely at home, as derived from the Patterns database, we could ascertain that organisations in census block groups 16.04-1 and 16.04-4 were subject to temporary quarantine. However, without more specific data on the accumulated incidence of coronavirus infections in individual fraternities and sororities, it was not possible to accurately isolate cases and controls.

### COVID-19 incidence versus visitation rates to the 68-restaurant comparison group

**Table A.** Case–control calculations for subset of bars and restaurants within 6-Minute walking distance of Sellery & Witte^a^^b^

Census block group	*N*	*b*	*r*
16.06-4 (1)	2392	177	115
16.06-3 (0)	1198	26	44
*R*		6.81	2.61

a Estimated odds ratio *R*_*b*_/*R*_*r*_ = 2.61 (95% CI 1.47–4.66).

b The walking time from Sellery-White to all bars in the entire 20-bar cluster ranged from 5 to 34 min. Four of the 20 bars were within a 6-minute radius. The walking time to restaurants in the entire 68-restaurant comparison group ranged from 3 to 13 min. 12 of the 68 restaurants were within a 6-minute radius.
